# Dual effects of VEGF-B on activating cardiomyocytes and cardiac stem cells to protect the heart against short- and long-term ischemia–reperfusion injury

**DOI:** 10.1186/s12967-016-0847-3

**Published:** 2016-05-04

**Authors:** Guo-hua Li, Bin Luo, Yan-xia Lv, Fei Zheng, Lu Wang, Meng-xi Wei, Xian-yu Li, Lei Zhang, Jia-ning Wang, Shi-you Chen, Jun-Ming Tang, Xiaohua He

**Affiliations:** School of Basic Medical Sciences, Wuhan University, Wuhan, 430071 Hubei Province China; Department of Cardiology and Institute of Clinical Medicine, Renmin Hospital, Hubei University of Medicine, Shiyan, Hubei Province 442000 China; Department of Physiology, School of Basic Medical Sciences, Hubei University of Medicine, Shiyan, Hubei Province 442000 China; Department of Pathophysiology, School of Basic Medical Sciences, Hubei University of Medicine, Shiyan, Hubei Province 442000 China; Department of Physiology and Pharmacology, University of Georgia, Athens, GA 30602 USA

**Keywords:** Cardiac stem cells, VEGF-B, Mobilization, Apoptosis, Angiogenesis

## Abstract

**Aims:**

To investigate whether vascular endothelial growth factor B (VEGF-B) improves myocardial survival and cardiac stem cell (CSC) function in the ischemia–reperfusion (I/R) heart and promotes CSC mobilization and angiogenesis.

**Methods and results:**

One hour after myocardial ischemia and infarction, rats were treated with recombinant human VEGF-B protein following 24 h or 7 days of myocardial reperfusion. Twenty-four hours after myocardial I/R, VEGF-B increased pAkt and Bcl-2 levels, reduced p-p38MAPK, LC3-II/I, beclin-1, CK, CK-MB and cTnt levels, triggered cardiomyocyte protection against I/R-induced autophagy and apoptosis, and contributed to the decrease of infarction size and the improvement of heart function during I/R. Simultaneously, an in vitro hypoxia-reoxygenation (H/R)-induced H9c2 cardiomyocyte injury model was used to mimic I/R injury model in vivo; in this model, VEGF-B decreased LDH release, blocked H/R-induced apoptosis by inhibiting cell autophagy, and these special effects could be abolished by the autophagy inducer, rapamycin. Mechanistically, VEGF-B markedly activated the Akt signaling pathway while slightly inhibiting p38MAPK, leading to the blockade of cell autophagy and thus protecting cardiomyocyte from H/R-induced activation of the intrinsic apoptotic pathway. Seven days after I/R, VEGF-B induced the expression of SDF-1α and HGF, resulting in the massive mobilization and homing of c-Kit positive cells, triggering further angiogenesis and vasculogenesis in the infracted heart and contributing to the improvement of I/R heart function.

**Conclusion:**

VEGF-B could contribute to a favorable short- and long-term prognosis for I/R via the dual manipulation of cardiomyocytes and CSCs.

**Electronic supplementary material:**

The online version of this article (doi:10.1186/s12967-016-0847-3) contains supplementary material, which is available to authorized users.

## Background

Ischemic heart disease (IHD) is the leading cause of death in the modern world. Myocardial infarction (MI) is usually initiated by myocardial ischemia resulting from the narrowing of coronary arteries due to atherosclerosis. At the clinical level, a number of cardiovascular intervention techniques, such as percutaneous coronary intervention (PCI) and cardiac bypass surgery, have been developed to restore myocardial perfusion [1]. Reperfusion occurs as a result of these interventions (particularly PCI); in short term, intervention should overcome ischemia–reperfusion injury (I/R), while in the long term, vascular restenosis should be effectively prevented via the activation of angiogenesis and vasculogenesis. However, the most effective therapeutic modality has not been clearly established.

The VEGF family of archetypal angiogenic growth factors consists of five secreted dimeric glycoprotein growth factors in mammals: VEGF (or VEGF-A), VEGF-B, VEGF-C, VEGF-D and PlGF (placenta growth factor) [2]. Although VEGF-mediated angiogenesis plays a critical role in the repair of ischemia/infarction, which is characterized by reduced blood supply to the heart [3, 4], until recently, VEGF-B was an exception to the family’s classical role in promoting angiogenesis [5, 6]. VEGF-B levels are highest in high metabolic tissues, such as the myocardium, skeletal muscle and adipose tissue [7, 8]. The absence of VEGF-B has been reported to lead to the decreased expression of fatty acid (FA) transport proteins in endothelial cells, which is associated with reduced lipid droplets in skeletal muscle and cardiomyocytes and improved insulin resistance in diabetes models [9–11]. VEGF-B is markedly down-regulated in the infarcted heart [12–14]. The over-expression of VEGF-B improved cardiac contractility in rats after experimental MI [15]. However, it is unknown whether VEGF-B plays a role in short- and long-term prognosis following myocardial ischemia for the development of heart failure.

In our previous study, cardiac stem cells (CSC), which are characterized by their migratory capabilities and potential for differentiating into cardiomyocytes and vascular cells, showed traits of VEGFR1 expression, were partly involved in VEGF-A-induced CSC migration, and contributed to the repair of the infarcted heart by inducing angiogenesis [3]. In addition to protecting cardiomyocytes, VEGF-B strongly induced myocardium-specific angiogenesis and arteriogenesis through VEGFR1 [5, 11, 15] and partly contributed to the involvement of endothelial cells in angiogenesis [11]. Of interest, CSCs are directly involved in the formation of the large coronary arteries [16]; thus, they predict the evolution of ischemic cardiomyopathy following revascularization [17]. At present, the relationship between VEGF-B-induced angiogenesis and CSCs during the process of myocardial ischemia or infarction, particularly in long-term I/R, is unknown. Angiogenic cytokine therapy has been widely regarded as an attractive approach for treating ischemic heart disease and for preventing vascular restenosis following cardiovascular intervention [1, 2, 4]. Therefore, we hypothesize that VEGF-B plays a pivotal role in the short- and long-term prognosis of myocardial I/R via the dual manipulation of cardiomyocytes and CSCs.

## Methods

All animal experiments were performed according to the *Guide for the Care and Use of Laboratory Animals* recommended by the US National Institutes of Health. All protocols for animal studies were permitted by the Institutional Animal Care and Use Committee of Hubei University of Medicine.

### Heart ischemia–reperfusion injury model

To observe whether VEGF-B protects against myocardial I/R injury in vivo, a rat model of myocardial I/R injury was established. Male Sprague–Dawley rats (240–280 g) were obtained from the Experimental Animal Center at Hubei University of Medicine and housed at an appropriate temperature (25 °C) with relative humidity (55 %), a fixed 12-h light/dark cycle and free access to food and water. The animals were randomly divided into four groups, as follows: a sham-operated group, an I/R injury group (I/R), a VEGF-B (1.0 μg/kg) group and a VEGF-B (10 μg/kg) group. The in vivo doses of VEGF-B were selected according to a previous study [18]. VEGF-B solution 200–300 μL (1.0 or 10 μg/mL) was injected with a 30-gauge tuberculin syringe into four sites (approximately 50–75 μL per site) into each I/R heart; volumes were determined according to the rat’s body weight. Two injection sites were in the myocardium bordering the ischemic area, and two were within the ischemic area. The animals were anesthetized with ketamine (50 mg/kg, ip) and xylazine (10 mg/kg, ip) and ventilated during left anterior descending coronary artery (LAD) ligation using a Colombus ventilator (HX-300, Taimeng Instruments, China). Surgery was performed under sterile conditions. The LAD was ligated for 1 h, and then opened for treatment with VEGF-B (local injection of the left myocardium, four sites at 50 μL per site) for 24 h or 7 days of reperfusion. In the sham-operation group, the rats underwent identical surgery but without ligation of the coronary artery. Buprenorphine hydrochloride (0.05 mg/kg, sc) was administered one time after the procedure.

### Measurement of creatine kinase (CK), CK-MB activity and cardiac troponin T (cTnT)

This procedure was described in detail elsewhere [19]. Briefly, 24 h after treatment, blood samples were centrifuged at 3500 rpm for 15 min at 4 °C; then the serum was collected. Subsequently, according to a handbook of experimental operations, CK activity (JianCheng Bioengineering Institute, Nanjing, China), CK-MB activity (Rapidbio, USA) and cardiac troponin T (cTnT) (Rapidbio, USA) levels, as enzymatic diagnostic indexes of myocardial injury, were detected and analyzed.

### Hemodynamic measurement

Hemodynamic measurement was performed as described previously [20]. Briefly, after 24 h of reperfusion, the animals were anesthetized with ketamine (50 mg/kg, ip) and xylazine (10 mg/kg, ip), and the left carotid artery was exposed. A catheter filled with heparinized (10 U/ml) saline solution was connected to a pressure transducer (Chengdu Taimeng Technology Co., Ltd., China) and then advanced into the left ventricle to record ventricular pressure for 15 min. Hemodynamic parameters were monitored simultaneously and recorded using Biological signal acquisition system BL-420S (Chengdu Taimeng Technology Co., Ltd., China).

### Histological measurement

Twenty-four hours after reperfusion, the hearts were removed and washed with K-H buffer at room temperature for 3 min, frozen at −20 °C for 1 h and transverse-sectioned into five parts (thickness, 2–5 mm). The sections were then incubated in 1 % 2, 3, 5-triphenyltetrazolium chloride (TTC, Sigma, USA) at 37 °C for 15 min. The infarcted myocardium was not stained by the TTC and appeared white in color; meanwhile, the non-ischemic myocardium was stained by the TTC and appeared brick-red in color. The infarction size was calculated by multiplying the planimetered areas by the slice thickness. The infarction size was expressed as the percentage of the left ventricular size of each heart.

### Cardiomyocyte apoptosis assay in vivo

To analyze cardiomyocyte apoptosis, 24 h after reperfusion, the hearts were removed, fixed in 4 % paraformaldehyde and embedded in an optimum cutting temperature compound (Fisher Scientific). Serial transverse Sections (5 μm) were cut across the longitudinal axis of the heart and mounted on slides. After a brief washing in phosphate-buffered saline (PBS), the heart sections were incubated in a blocking buffer [PBS containing 1 % fetal calf serum (FCS) and 0.1 % Triton X-100] at room temperature for 1 h. Cardiomyocyte apoptosis was detected using the methods described in the manual of the In-Situ Apoptosis Detection Kit (MM_NF-S7165#, Millipore, UEA). Five fields from each section (n = 3) were randomly selected. The number of apoptotic cells was counted manually by two pathologists, who were unaware of the experimental design. The rate of apoptosis was calculated as the percentage of all cells per high power field (HPF; 400×).

### Immunostaining

Seven days after I/R, the heart tissues were fixed in 4 % paraformaldehyde and embedded in optimum cutting temperature compound (Fisher Scientific). Serial transverse Sections (5 μm) were cut across the longitudinal axis of the heart and washed with phosphate-buffered saline (PBS). The heart sections were then incubated in a blocking buffer at room temperature for 1 h. The sections were incubated in antibodies (diluted 1:250 in blocking buffer) at 4 °C overnight for primary antibodies and at room temperature for 2 h for secondary antibodies. The primary antibodies used were mouse anti-rat vWF VIII, rabbit anti-rat a-SMA (Santa Cruz) and anti-rat c-kit (Abcam). The secondary antibodies were FITC-conjugated anti-rabbit IgG, FITC-conjugated anti-rat IgG, and FITC-conjugated anti-mouse IgG (Santa Cruz) [3]. Images of c-Kit positive cells or vessels were acquired under microscopy (magnification, 400×) for five random fields in the infarcted area of each transverse slice for all three groups. The number of c-Kit positive cells or vessels in the infarcted areas was counted manually by two pathologists who were unaware of the experimental design and are presented as the mean of c-Kit positive cells or blood vessels per unit area (0.2 mm^2^).

### Culture of H9c2 rat cardiomyocytes

The rat embryonic ventricular myocardial cell line H9c2 was purchased from the Cell Bank of the Chinese Academy of Sciences (Shanghai, China). H9c2 cells were cultured as described previously [19].

### Hypoxia-reoxygenation of H9c2 Cells

The in vitro hypoxia-reoxygenation of H9c2 cells occurred as described previously [19]. In brief, H9c2 cells were treated with or without VEGF-B (20 ng/ml, Peprotech, USA) in EBSS after H9c2 cells culture under low-oxygen conditions (95 % N_2_  +  5 % CO_2_) for 6 h in a humidified hypoxia chamber (Stem Cell Technology, USA). The in vitro doses of VEGF-B (0.1, 1, 10, 20 and 50 ng/ml) were determined as described in detail elsewhere [21]. After hypoxic incubation, the medium was replaced with 15 % FBS medium under normoxic conditions (95 % air  +  5 % CO_2_) for reoxygenation for 3 h.

### Cell apoptosis assay in vitro

Cell apoptosis was determined as described previously [19]. According to the cell apoptosis protocol, H9c2 cells were resuspended in 500 µl binding buffer after washing and then incubated with Annexin V solution for 3 min, followed by PI solution (Bender MedSystems, Austria) for 15 min. The apoptotic cells were analyzed with flow cytometry.

### Lactate dehydrogenase (LDH) release

The LDH level is an indicator of cellular injury [19]. The LDH release was measured using commercial kits (JianCheng Bioengineering Institute, China).

### Cell autophagy assay

H9c2 cells were cultured on a chamber slide to 50–60 % confluence. The cells were transfected with Ad-mRFP-GFP-LC3-I/II (MOI: 100, Hanbio, Co, China) for 48 h and then treated with VEGF-B (10 ng/ml) according the protocol for the hypoxia-reoxygenation of H9c2 Cells.

### Western blot

The H9c2 cells or heart tissues were lysed in RIPA buffer, and 30 µg of proteins were separated in a 12 % SDS–PAGE gel and transferred onto a nitrocellulose membrane (Millipore). The membrane was rinsed with Tris-buffered saline (TBS) containing 0.1 % Tween-20 (TBST) and blocked with 5 % fat free milk in TBS at room temperature for 1 h. The membrane was then incubated with anti-Akt, anti-phospho-Akt, anti-Bcl-2, anti-Bax, anti-beclin1, anti-p38MAPK, anti-ERK1/2, anti-pp38MAPK, anti-pERK1/2 (Cell Signaling Technology, 1:500), anti-SDF-1α, anti-HGF (Santa Cruz) or anti-α-Tubulin antibody (Sigma, 1:5000) and then incubated with horseradish peroxidase-conjugated secondary antibodies (1:10,000 dilution; Santa Cruz). The immunoblots were detected using enhanced chemiluminescence reaction (Amersham Pharmacia Biotech) and measured with densitometry.

### Statistical analysis

All data are expressed as mean ± SD. The parameters of two groups were compared using the unpaired t test. The parameters of more than two groups were compared using one-way analysis of variance. *P* values <0.05 were considered statistically significant.

## Results

### VEGF-B reduced infarct size and improved heart function

**Fig. 1:**
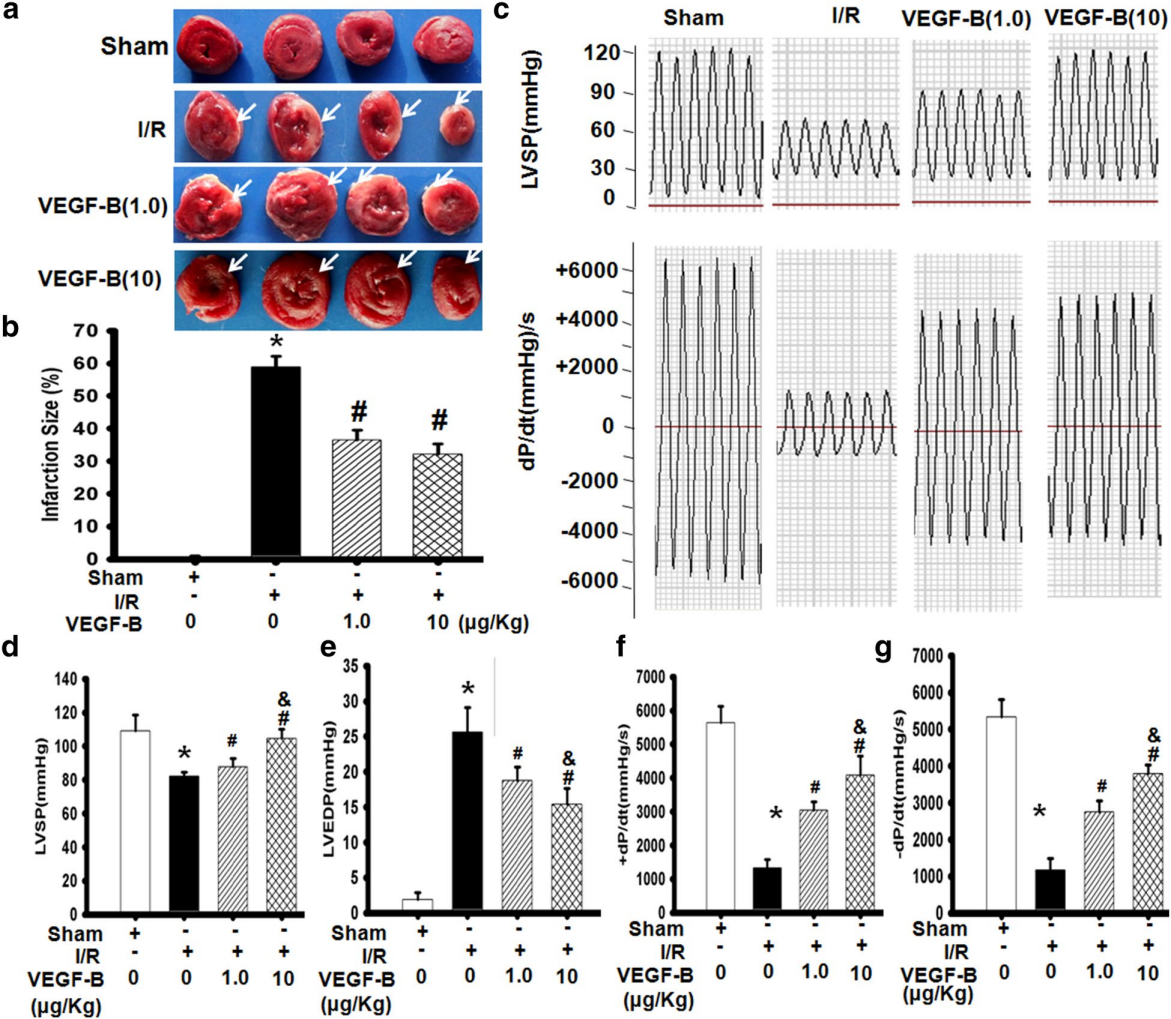
VEGF-B improved heart function in a myocardium I/R model. **a** TTC-stained myocardium 24 h after reperfusion. The *white arrows* indicate the infarct area of heart. **b** Infarction size in each group. The infarct size was expressed as a percentage of the *left* ventricular size for each heart. *P < 0.01 vs. sham group; ^#^ *P* < 0.01 vs. I/R group, n = 6. **c** Typical image of heart function. 24 h after reperfusion, LV function was measured as described in the “Methods” section. LVSP-left ventricle systolic pressure (**d**); LVEDP-left ventricle end-diastolic pressure (**e**); ±d*p*/d*t*_max_-rate of the rise or fall of *left* ventricular pressure (**g** and **h**). *P < 0.01 vs. sham group; ^#^ *P* < 0.05 vs. I/R group; ^&^ *P* < 0.05 vs. VEGF-B (1.0) group, n = 6

To observe the effect of VEGF-B on heart I/R injury in vivo, we performed I/R experiments that mimic therapeutic reperfusion in human patients. Rat hearts were subjected to ischemia by ligating the LAD for 1 h, followed by reperfusion for 24 h to assess the effect of VEGF-B on myocardial infarct size and heart function in an I/R model in vivo. One-percent TTC staining was used to evaluate the infarct size of rat hearts exposed to reperfusion with or without VEGF-B treatment. The results showed that 1.0 and 10 μg/kg VEGF-B treatment significantly reduced the infarct size in vivo compared with the I/R group. Furthermore, the 10 μg/kg VEGF-B group showed a greater decrease in the infarct size compared with the 1.0 μg/kg VEGF-B group (Fig. 1a, b). In line with the change in infarct size, the left ventricle function of the heart in both VEGF-B groups was significantly improved compared with the I/R group. Furthermore, the higher dose of VEGF-B had a much greater effect on increasing LVSP and ±dp/dt_max_ and decreasing LVEDP compared with the lower dose of VEGF-B (Fig. 1c–f).

### VEGF-B suppressed CK, CK-MB activity, and cTnT levels on heart I/R injury

To monitor myocardial damage, CK, CK-MB, and cTnT activity was detected to evaluate myocardial injury. The results showed that CK, CK-MB, and cTnT activity were obviously increased in the I/R group compared with the sham group (Additional file 1: Figure S1A–D). Interestingly, compared with the I/R group, the VEGF-B groups all had significantly reduced CK, CK-MB activity and cTnT levels. Furthermore, high-dose VEGF-B had better protective effects against heart I/R (Additional file 1: Figure S1A–C). These data suggest that VEGF-B inhibits I/R-mediated myocardial injury.

### VEGF-B inhibits I/R-mediated cardiocyte apoptosis in vivo

**Fig. 2:**
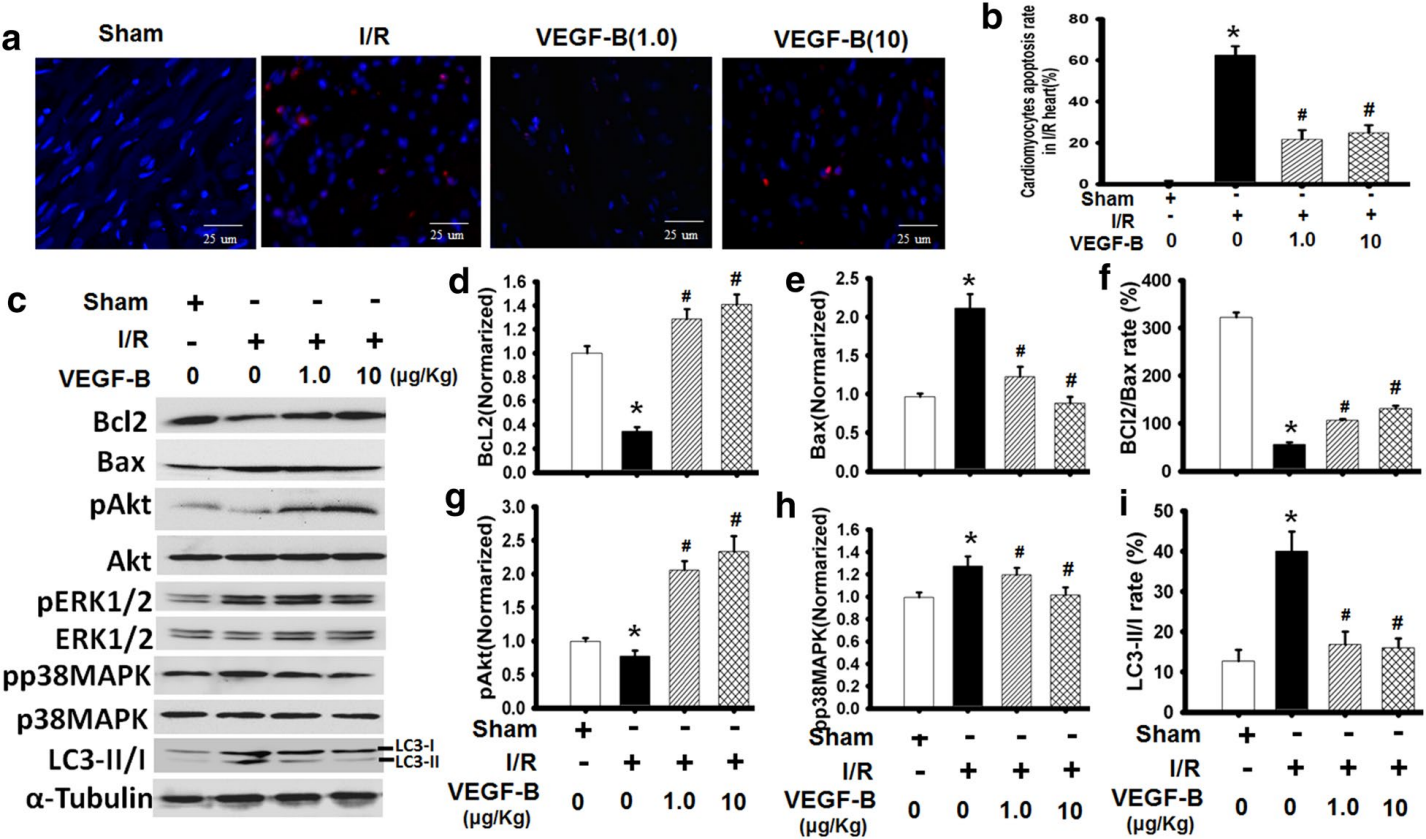
VEGF-B inhibited I/R-mediated cardiocyte apoptosis in vivo. **a** A typical image of cardiomyocyte apoptosis in the heart I/R model was detected and analyzed using the TUNNEL method. *Red fluorescence* apoptotic cells; *Blue fluorescence* cell nucleus. **b** Quantitative analysis of the cell apoptosis rate in HPF. *P < 0.01 vs. sham group; ^#^ *P* < 0.05 vs. I/R group, n = 6. **c** Bcl-2, pAkt, Bax, pERK1, pp38MAPK and LC3-II/I expression was detected using western blot, as described in the Materials and methods section (**c**), and quantified using normalization to a-tubulin (**d**–**i**). *P < 0.01 vs. sham group; ^#^ *P* < 0.05 vs. I/R group, n = 6

To further assess the potential protective mechanism of VEGF-B in heart I/R injury, cardiomyocyte apoptosis, a classical marker of myocardial I/R damage, was examined after VEGF-B treatment. We found that VEGF-B significantly decreased the apoptosis rate of cardiocytes in the in vivo I/R model (Fig. 2a, b). Bcl-2/Bax, two typical indicators of cardiocyte apoptosis, were used to evaluate the effect of VEGF-B on myocardium exposed to I/R. The results showed that Bcl-2 expression was obviously induced and Bax expression was significantly decreased in VEGF-B-treated hearts compared with the I/R group (*P* < 0.05). Finally, Bcl-2/Bax regulation was generally involved in multiple signaling pathways, such as PI3K/Akt, ERK1/2 and p38MAPK, et al. [22]. The changes in nonphosphorylated and phosphorylated Akt, ERK1/2 and p38MAPK were analyzed and compared with those of the I/R group. Akt, ERK1/2 and p38MAPK and pERK1/2 levels were not markedly increased (Additional file 1: Figure S2), but pAkt levels were obviously increased in both the VEGF-B (1.0) and VEGF-B (10) group (Fig. 2c–e). Meanwhile, p-p38MAPK levels were decreased in the two VEGF-B groups. Furthermore, the higher dose of VEGF-B had better benefits for increasing pAkt levels and decreasing p-ERK1/2 and p-p38MAPK levels.

In addition, because VEGF-B is a newly identified metabolic factor [23] associated with cell autophagy, we also examined changes in LC3-II/I, an indicator of autophagy. We found that the LC3-II/I rates were increased in I/R group compared to with the sham group and that compared with the I/R group, the two VEGF-B treatment groups showed decreased LC3-II/I rates, and the higher dose of VEGF-B had a better effect on reducing LC3- II/I rates (Fig. 2c, i).

These data suggest that VEGF-B could prevent I/R-mediated cardiocyte autophagy and apoptosis associated with the PI3K/Akt and p38MAPK signaling pathways.

### VEGF-B reversed the changes in H/R-induced H9c2 cell apoptosis in vitro

**Fig. 3:**
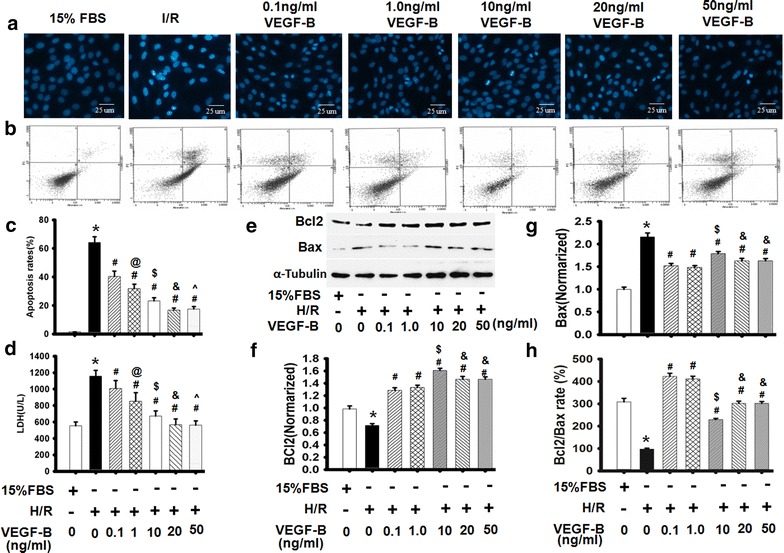
VEGF-B restored H/R-induced alterations of H9C2 cell morphology and cell apoptosis. **a** Cell apoptosis was detected using DAPI. **b** Cell apoptosis was detected using Annexin V/PI staining, n = 6. **c** Quantitative analysis of cell apoptosis in (**b**). *P < 0.05 vs. 15 % FBS-treated cells; ^#^ *P* < 0.05 vs. H/R-treated cells; ^@^ *P* < 0.05 vs. 0.1 ng/ml VEGF-B-treated cells; ^$^ *P* < 0.05 vs. 1.0 ng/ml VEGF-B-treated cells; ^&^ *P* < 0.05 vs. 10 ng/ml VEGF-B-treated cells; ^^^ *P* > 0.05 vs. 20 ng/ml VEGF-B-treated cells, n = 6. **d** VEGF-B reduced LDH levels. *P < 0.05 vs. 15 % FBS-treated cells; ^#^ *P* < 0.05 vs. H/R-treated cells; ^@^ *P* < 0.05 vs. 0.1 ng/ml VEGF-B-treated cells; ^$^ *P* < 0.05 vs. 1.0 ng/ml VEGF-B-treated cells; ^&^ *P* < 0.05 vs. 10 ng/ml VEGF-B-treated cells; ^^^ *P* > 0.05 vs. 20 ng/ml VEGF-B-treated cells, n = 6. **e** Bcl-2 and Bax expression was detected using western blot, as described in the Materials and methods section (**e**), and quantified via normalization to a-tubulin (f–h). *P < 0.05 vs. 15 % FBS-treated cells; ^#^ *P* < 0.05 vs. H/R-treated cells; ^$^ *P* < 0.05 vs. 1.0 ng/ml VEGF-B-treated cells; ^&^ *P* < 0.05 vs. 10 ng/ml VEGF-B-treated cells, n = 6

**Fig. 4:**
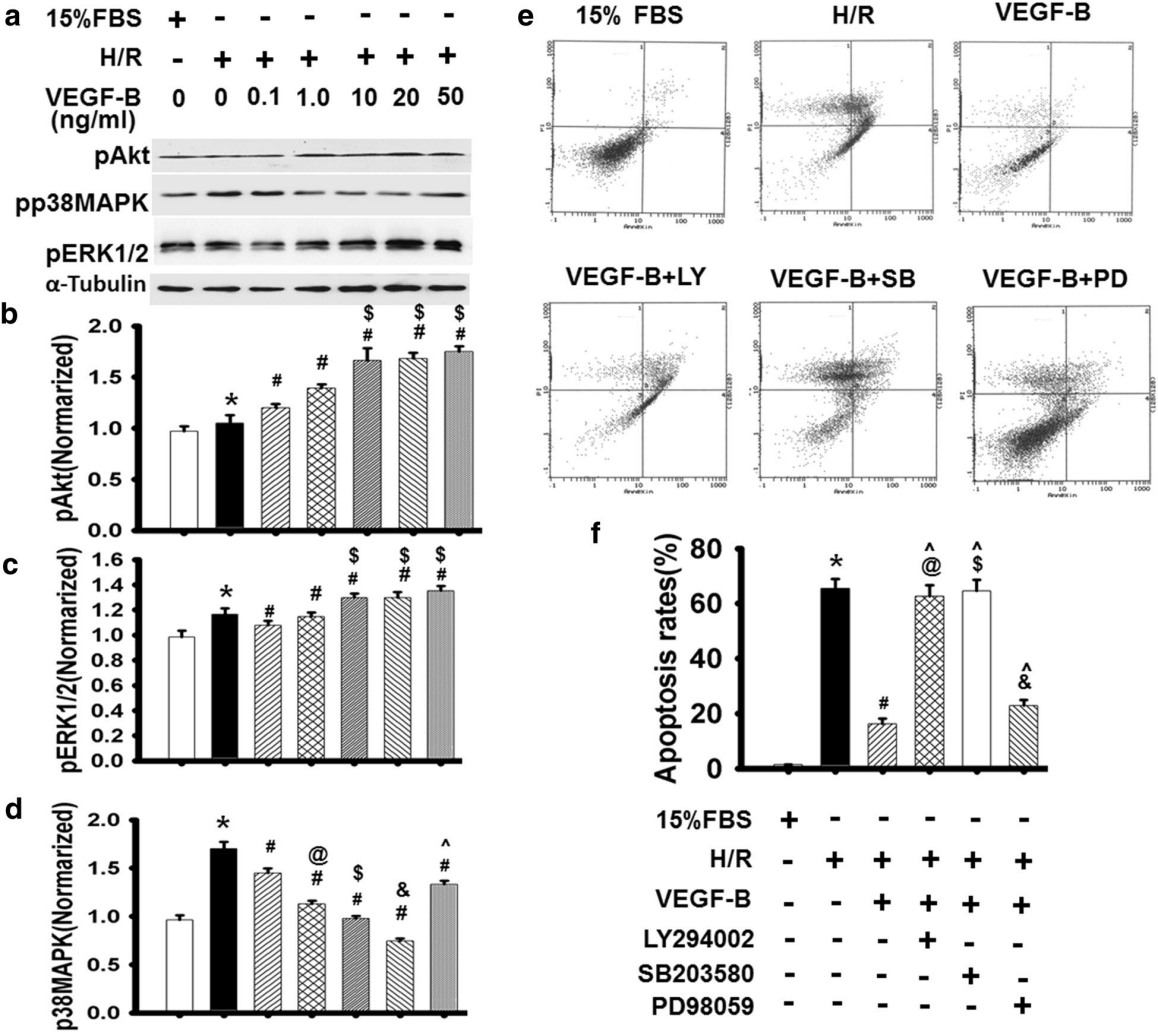
VEGF-B inhibited H/R-induced H9c2 cell apoptosis in vitro. **a** pAkt, pERK1 and pp38MAPK expression was detected using western blot, as described in the “Methods” section (**b**), and quantified via normalization to a-tubulin (**b**–**d**). *P < 0.05 vs. 15 % FBS-treated cells; ^#^ *P* < 0.05 vs. H/R-treated cells; ^@^ *P* < 0.05 vs. 0.1 ng/ml VEGF-B-treated cells; ^$^ *P* < 0.05 vs. 1.0 ng/ml VEGF-B-treated cells; ^&^ *P* < 0.05 vs. 10 ng/ml VEGF-B-treated cells; ^^^ *P* < 0.05 vs. 20 ng/ml VEGF-B-treated cells, n = 6. **e** Cell apoptosis was detected using Annexin V/PI staining, n = 6. **f** Quantitative analysis of cell apoptosis in (**e**). *P < 0.05 vs. 15 % FBS-treated cells; ^#^ *P* < 0.05 vs. H/R-treated cells; ^@^ *P* > 0.05 vs. H/R-treated cells; ^$^ *P* > 0.05 vs. H/R-treated cells; ^&^ *P* < 0.05 vs. H/R-treated cells; ^^^ *P* < 0.05 vs. 20 ng/ml VEGF-B-treated cells, n = 6

To develop a detailed description of the role and mechanism of VEGF-B in I/R injury, an in vitro H/R-induced H9c2 cell damage model was established to mimic the in vivo myocardial I/R model. DAPI staining showed that H/R injury caused significant apoptosis in H9c2 cells, as shown by condensed nuclei, an indicator of apoptosis. However, VEGF-B restored H9c2 nuclei to their normal morphology (Fig. 3a). The appearance of phosphatidylserine (PS) in the outer leaflet of the phospholipid bilayer without a disruption in membrane integrity is one of the earliest characteristics of apoptotic cells [20]. Flow cytometry assay was used to evaluate the changes in cell apoptosis using Annexin V/PI staining, which indicates PS production. As Fig. 4b shows, I/R induced H9c2 cell apoptosis. However, VEGF-B attenuated the number of Annexin V/PI-positive cells, suggesting that VEGF-B inhibited H/R-induced apoptosis (Fig. 3b). Furthermore, the protective effects of VEGF-B on H9c2 cells exposed to H/R showed dose-dependent characteristics (Fig. 3c).

LDH release is an indicator of cellular injury [22]. Compared with untreated cells, LDH levels were markedly increased by H/R injury. VEGF-B treatment, however, decreased H/R-induced LDH release (Fig. 3d). Meanwhile, H/R obviously induced Bax expression and decreased Bcl-2 expression in H9c2 cells, and VEGF-B treatment significantly increased Bcl-2 expression while reducing Bax expression in H/R-injured H9c2 cells in a dose-dependent manner (Fig. 3e–h). These data suggest that VEGF-B is able to protect cardiomyocytes from H/R-induced injury in a dose-dependent manner.

### VEGF-B regulated cell apoptosis in vitro

A recent publication indicates that apoptosis is mediated by numerous signaling pathways, such as PI3K/Akt, ERK/1/2 and p38 MAPK [23]. To test whether these mechanisms apply to VEGF-B-induced myocardium protection, we used VEGF-B to stimulate cardiomyocytes before H/R injury. We found that VEGF-B obviously enhanced pAkt, slightly increased pERK1/2, and decreased p-p38MAPK without affecting their expression (Additional file 1: Figure S3) in a dose-dependent manner (Fig. 4a–d). To confirm whether these signaling molecules were involved in VEGF-B-mediated anti-apoptosis, special inhibitors were used to assess the relationship between them. The results showed that the ERK1/2 inhibitor PD98059 did not obviously abolish the anti-apoptotic effect of VEGF-B, but the p38MPAK inhibitor SB203580 and the PI3K specific inhibitor LY294002 obviously blocked it (Fig. 4e, f), suggesting that VEGF-B prevents apoptosis in cardiomyocytes through PI3K/Akt and p38MAPK signaling.

### VEGF-B inhibited H/R-induced H9c2 cell apoptosis through the regulation of Bcl-2/Bax expression mediated by PI3K/Akt signaling

Because the cell survival regulators Bcl-2 and Bax are regulated by PI3K/Akt signaling [23] and VEGF-B had a regulatory effect on Bcl-2/Bax expression (Fig. 3e–h), we examined whether VEGF-B-mediated Bcl-2/Bax regulation was involved in PI3K/Akt signaling. As shown in Additional file 1: Figure S4A, VEGF-B markedly increased Bcl-2 while decreasing Bax expression, suggesting that VEGF-B prevents apoptosis in H9c2 cells by readjusting the expression and ratio of Bcl-2 and Bax (Additional file 1: Figure S4A). LY294002 blocked VEGF-B-mediated Bcl-2 and Bax expression (Additional file 1: Figure S4A, 4D–4F), suggesting that VEGF-B regulates Bcl-2/Bax expression through the PI3K/Akt signaling pathway.

### VEGF-B inhibited I/R-induced autophagy in a dose-dependent manner

**Fig. 5:**
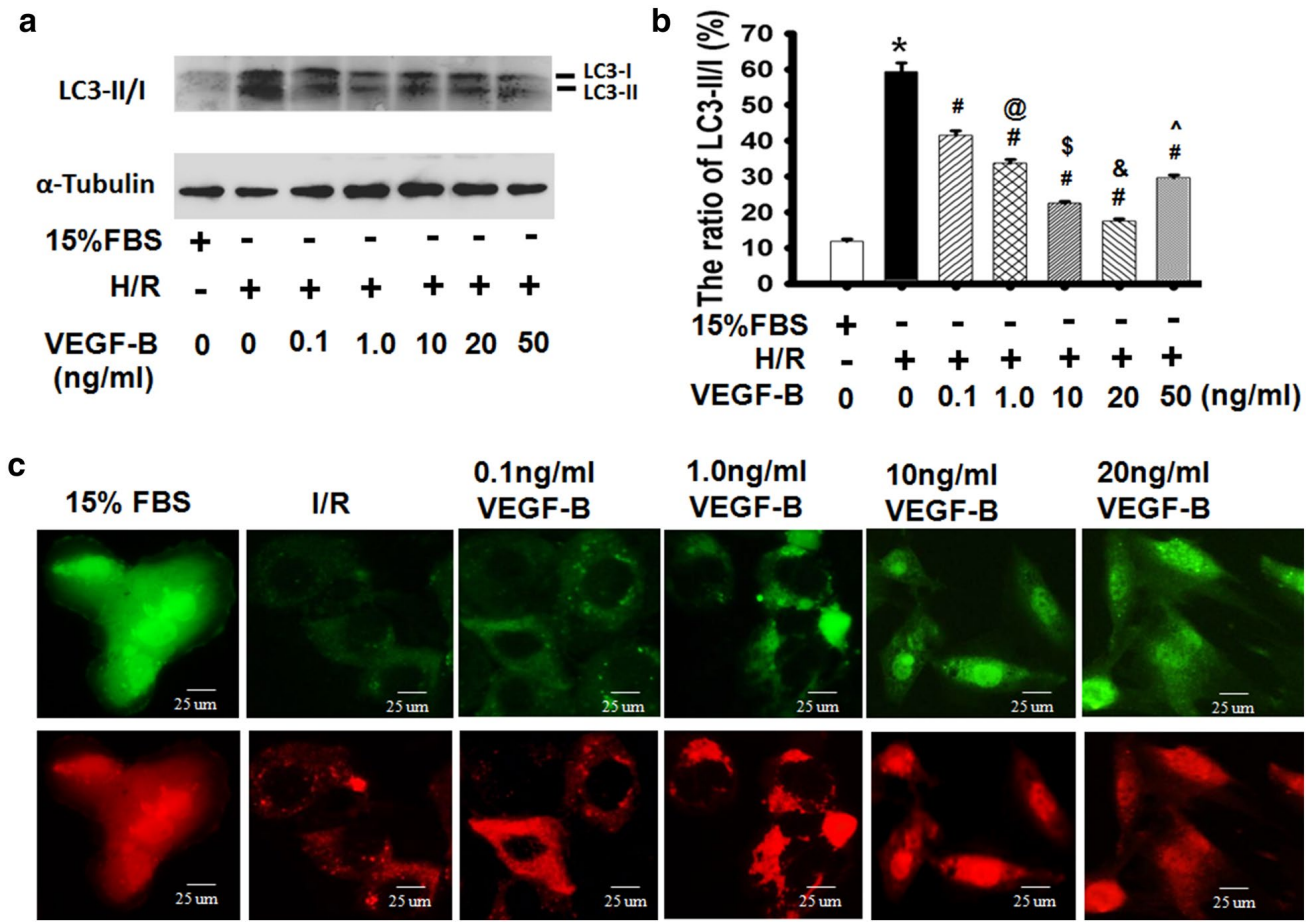
VEGF-B inhibited H/R-induced autophagy. **a** LC3-II/I expression was detected using western blot, as described in the “Methods” section (**a**), and quantified via normalization to a-tubulin (**b**). *P < 0.05 vs. 15 % FBS-treated cells; ^#^ *P* < 0.05 vs. H/R-treated cells; ^@^ *P* < 0.05 vs. 0.1 ng/ml VEGF-B-treated cells; ^$^ *P* < 0.05 vs. 1.0 ng/ml VEGF-B-treated cells; ^&^ *P* < 0.05 vs. 10 ng/ml VEGF-B-treated cells; ^^^ *P* < 0.05 vs. 20 ng/ml VEGF-B-treated cells, n = 3. **c** Representative images showing LC3 staining in different groups of H9c2 cells infected with Ad-GFP-RFP-LC3 for 48 h

VEGF-B is a newly identified metabolic factor [24] that could be associated with cell autophagy. To determine whether VEGF-B affected autophagy, as shown in Fig. 5a, H/R increased the expression of autophagy-related proteins in H9c2 cells. VEGF-B reduced the levels of autophagy-related proteins and the ratio of LC3-II/I seen on western blot analysis (Fig. 5a, b). The GFP-RFP-LC3 adenovirus (Ad-mRFP-GFP-LC3) system was further used to confirm the induction of autophagy using punctate forms, which represent autophagosome formation [25]. As Fig. 3c shows, after infection with Ad-mRFP-GFP-LC3, we observed the successful introduction of this adenovirus and both fluorescent proteins. In addition to accumulation of LC3, there were more red puncta in the H/R-induced H9c2 cells than in the control cells. Furthermore, VEGF-B markedly decreased the number of red puncta in a dose-dependent manner. These results further confirmed the induction of autolysosome formations in the H/R model, indicating that VEGF-B mediated an anti-autophagy flux in H9c2 cells.

### VEGF-B inhibited H9c2 cell apoptosis by inhibiting H/R-induced autophagy

**Fig. 6:**
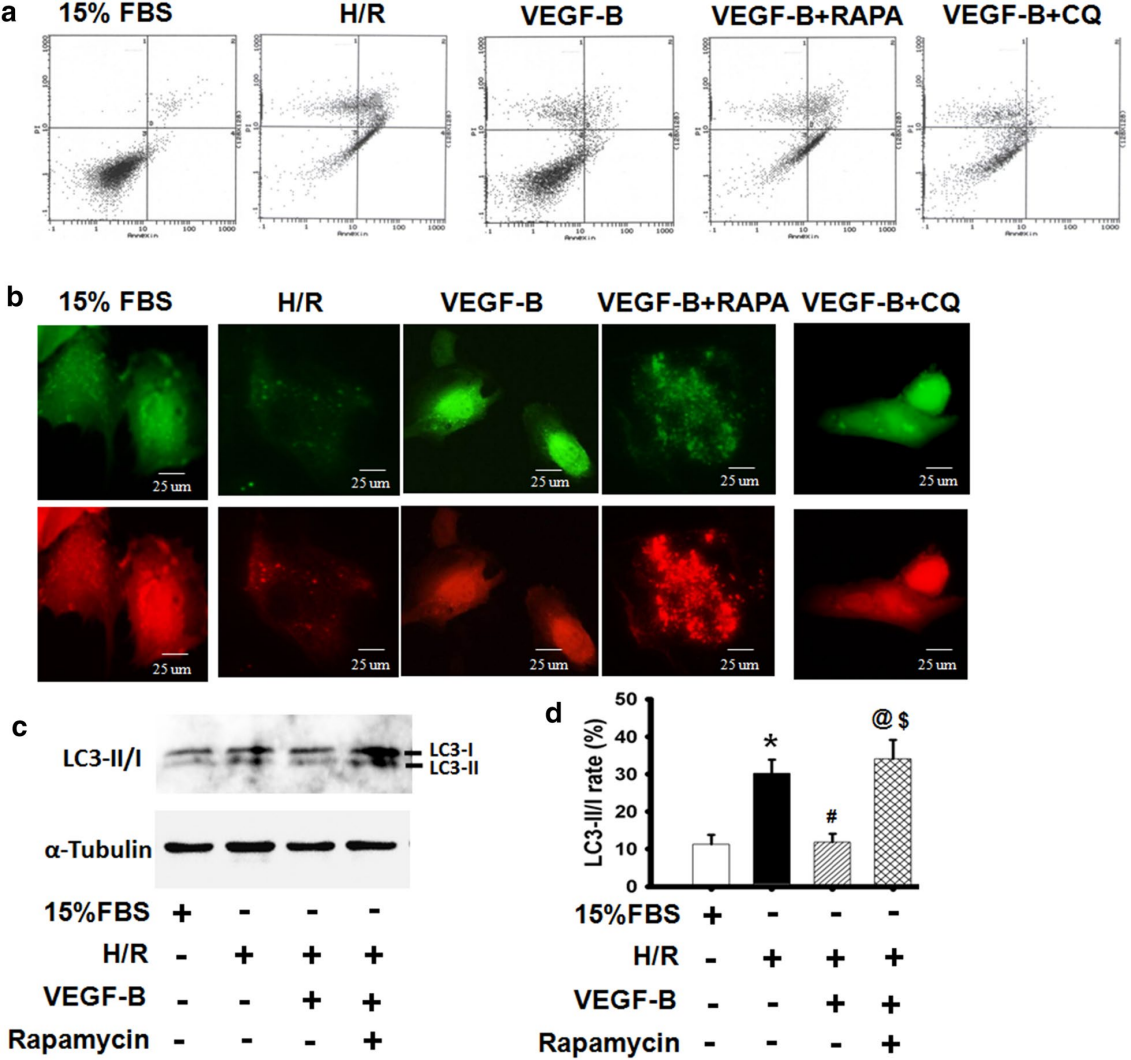
VEGF-B inhibited H9c2 cell apoptosis by inhibiting H/R-induced autophagy. **a** Cell apoptosis was detected using Annexin V/PI staining, n = 6. **b** Quantitative analysis of cell apoptosis in (**a**). **c** Representative images showing LC3 staining in different groups of H9c2 cells infected with Ad-GFP-RFP-LC3 for 48 h. **c** LC3-II/I expression was detected using western blot, as described in the “Methods” section (**a**), and quantified using normalization to a-tubulin (**d**). *P < 0.05 vs. 15 % FBS-treated cells; ^#^ *P* < 0.05 vs. H/R-treated cells; ^@^ *P* < 0.05 vs. 20 ng/ml VEGF-B-treated cells; ^$^ *P* > 0.05 vs. H/R-treated cells, n = 6

To further explore the involvement of VEGF-B in H9c2 cell apoptosis in the H/R-induced autophagy model, the autophagy inducer rapamycin and the autophagy inhibitor chloroquine were used to determine whether VEGF-B’s effect on autophagy involved H9c2 cell apoptosis. As Fig. 6a and c show, rapamycin reversed the VEGF-B-mediated anti-apoptosis effects, the VEGF-B-mediated anti-autophagy effects and the LC3-II/I rates in the H/R model (Fig. 6d, e), suggesting that VEGF-B-mediated anti-autophagy plays a critical role in the protective effects of VEGF-B in H9c2 cells under H/R conditions. Additionally, chloroquine enhanced the VEGF-B-mediated anti-autophagy and anti-apoptosis effects in H/R-induced cell injury. These data demonstrate that VEGF-B could inhibit H/R-induced cell apoptosis by blocking autophagy.

### VEGF-B inhibited autophagy-mediated H9c2 cell apoptosis

**Fig. 7:**
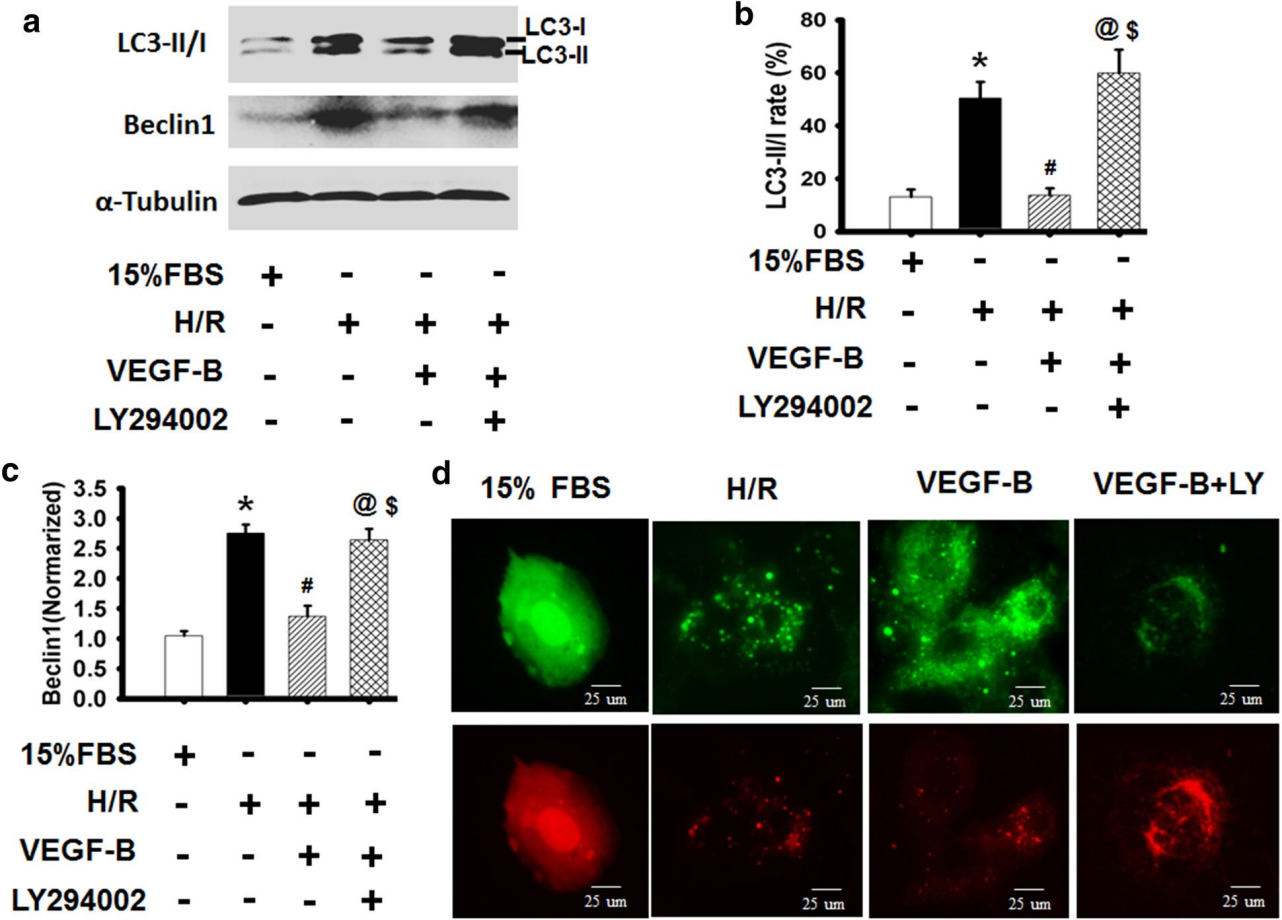
VEGF-B inhibited H/R-induced autophagy through PI3/Akt signaling. **a** LC3-II/I expression was detected using western blot, as described in the “Methods” section (**a**), and quantified via normalization to a-tubulin (**b**, **c**). *P < 0.05 vs. 15 % FBS-treated cells; ^#^ *P* < 0.05 vs. H/R-treated cells; ^@^ *P* < 0.05 vs. 20 ng/ml VEGF-B-treated cells; ^$^ *P* > 0.05 vs. H/R-treated cells, n = 6. **d** Representative images showing LC3 staining in different groups of H9c2 cells infected with Ad-GFP-RFP-LC3 for 48 h, n = 6

Because the p38MAPK and PI3K/Akt signaling pathways play important roles in both apoptosis and autophagy [26, 27], the PI3K-specific inhibitor LY294002, an autophagy inducer, was used to observe the effects of VEGF-B on H9c2 cells under H/R conditions [27]. As Fig. 7a, b show, the expression of autophagy-related proteins and LC3-II/I rates increased and the autophagy flux (shown in red) was enhanced in H/R-induced injury of H9c2 cells. VEGF-B reduced the levels of autophagy-related proteins and LC3-II/I rates, and the special effects of VEGF-B on autophagy could be abolished by the PI3K-specific inhibitor LY294002, suggesting that VEGF-B inhibited autophagy via PI3K/Akt signaling (Fig. 7a–d). Interestingly, the special effects of VEGF-B on anti-autophagy could not be abolished by the ERK1/2 inhibitor PD98059 but could be abolished by the p38MPAK inhibitor SB203580 (Additional file 1: Figure S5), which was consistent with the finding that PD98059 and not SB203580 inversed VEGF-B-mediated anti-apoptosis (Fig. 5e). These results imply that VEGF-B can inhibit autophagy-mediated H9c2 cell apoptosis by regulating the PI3K/Akt, ERK1/2 and p38MPAK signaling pathways.

### VEGF-B promoted CSC mobilization, angiogenesis and improved heart function

**Fig. 8:**
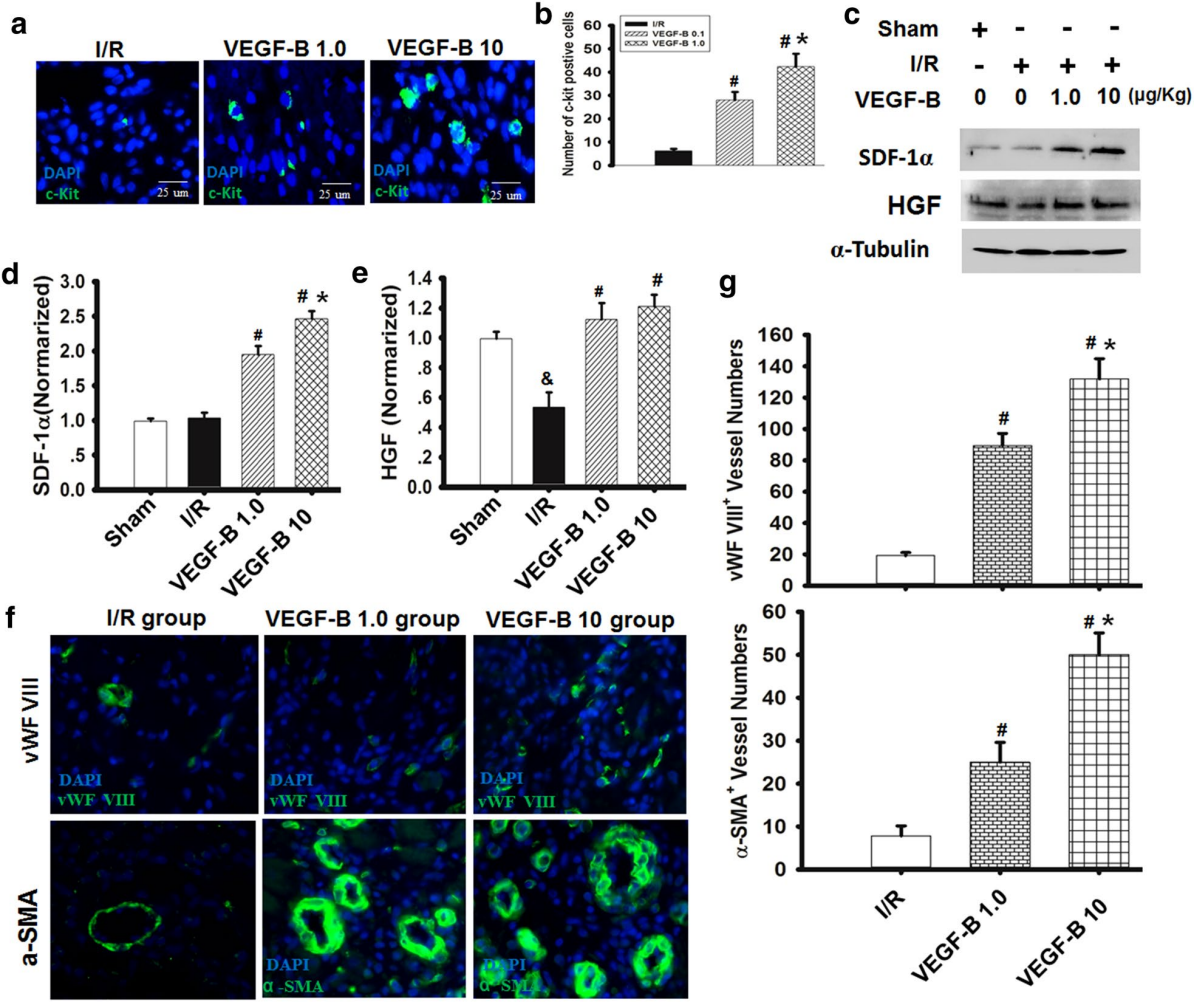
VEGF-B promoted CSC mobilization and angiogenesis and improved heart function. **a**, **b** The typical picture of c-kit-positive CSCs in I/R hearts after VEGF-B treatment. Blue, DAPI-labeled nucleus; green, c-kit positive. **b** The number of c-kit-positive CSCs in I/R hearts was counted, and statistical analyses were performed. The *bar* represents 25 µm. ^#^ *P* = 0.05 vs. I/R group; *P < 0.05 vs. VEGF-B (1.0) group, n = 6. **c**–**e** SDF-1α and HGF expression was detected using western blot, as described in the “Methods” section (**c**), and quantified via normalization to a-tubulin (**d**, **e**). n = 6. ^#^ *P* < 0.05 vs. I/R group; *P < 0.01. vs. VEGF-B (1.0) group; ^&^ *P* < 0.01. vs. sham group. **f** Typical picture of vWF VIII and a-SMA immunostaining for the analysis of vessel density in the infarcted heart. **g** Quantitative analysis of vessel density, as indicated. ^#^ *P* < 0.05 vs. I/R group; *P < 0.01. vs. VEGF-B (1.0) group, n = 6

To determine whether VEGF-B can stimulate the mobilization of CSC in infarcted hearts, we locally injected VEGF-B into the heart prior to LAD ligation, isolated the infarcted myocardium 7 days after I/R, and detected the presence of CSC by immunostaining stem cell markers for c-kit. A large number of c-kit-positive cells were observed in the VEGF-B-treated myocardium (Fig. 8a, b). Because SDF-1α and HGF play important roles in CSC mobilization in the infarcted myocardium, we detected SDF-1α and HGF expression in the infarcted heart using western blotting and found that SDF-1α and HGF expression obviously increased in infarcted myocardium treated with VEGF-B in a dose-dependent manner (Fig. 8c–e).

CSCs are closely related to the differentiation of vascular cells, such as endothelial cells and vascular smooth muscle cells [16]. Consequently, 7 days after VEGF-B treatment, angiogenesis in the I/R heart was evaluated according to microvessel density and artery vessel density, as indicated by the expression of vWF VIII, a mature endothelial cell marker, and α-SMA, a vascular smooth muscle cell marker, respectively (Fig. 8f). We found that VEGF-B significantly induced blood vessel formation in dose-dependent manner, resulting in increased vessel density (Fig. 8g). An in vitro tube formation assay of SDF-1α receptor/CXCR4 inhibitor AMD3100 and the HGF receptor c-Met inhibitor SU11274 were used to further confirm whether VEGF-B-induced angiogenesis involved SDF-1α and HGF (Additional file 2). As shown in Additional file 1: Figure S6, CM-VB induced the formation of new tubes from c-kit-positive cells in vitro, and the effect could be partly abolished by AMD3100 or SU11274. These data demonstrate that VEGF-B induced angiogenesis mediated by c-kit-positive cells in infarcted myocardium by activating SDF-1α and HGF signaling.

Furthermore, VEGF-B treatment significantly improved the function of the left ventricle of the heart, and the higher dose of VEGF-B had a much greater effect on increasing LVSP and ±dp/dt_max_ and decreasing LVEDP compared with the lower dose (Additional file 1: Figure S7A–D).

## Discussion

In this study, we demonstrate for the first time that human VEGF-B protein promotes cardiomyocyte survival by activating the PI3K/Akt/Bcl-2/Beclin1 signaling pathway after short-term I/R and activates CSC mobilization and angiogenesis via the induced expression of SDF-1 and HGF in infarcted heart after long-term I/R. We also demonstrated improvement in cardiac function following VEGF-B treatment.

Previous studies demonstrated that VEGF-B had a protective role in the heart after experimental M) or angiotensin II-induced heart failure in rats [5, 28–31]. The transgene (TG), adenoviral-mediated (Ad) or adeno-associated virus (AAV) expression of VEGF-B used in the animal model of MI or heart failure is strong and long-term, and the safety and controllability of these vectors are controversial for practical application [32]. Clinically, human VEGF-B protein should be more far more acceptable to patients. Recombinant human VEGF-B protein were therefore used in the present study to determine the beneficial effects of VEGF-B on cardiomyocyte protection, CSC mobilization, angiogenesis and cardiac function of I/R rats.

A recent study shows that VEGF-B induces compensatory hypertrophy and preserves cardiac function through VEGFR-1 activation of cardiomyocytes after MI [15]. Pathways downstream of VEGF-B/VEGFR1, including the Akt/mTORC1 and ERK1/2, p38MAPK pathways, which are known to be associated with cardiomyocyte growth and arteriogenesis, were activated in the heart [31, 33, 34]. Consequently, VEGF-B’s protective effect on cardiomyocytes is dependent on PI3K/Akt signaling, which is in line with the pro-survival function of VEGF-A [35, 36]. The anti-apoptotic effects of VEGF-B may be interpreted using the PI3K inhibitor LY294002 and the ERK1/2 inhibitor PD98059 to abolish its roles. The unexpected outcome is that the p38MAPK blocker SB203580 inversed the anti-apoptotic effects of VEGF-B on H/R- induced H9c2 cells, while VEGF-B decreased the levels of p-p38MAPK induced by H/R, indicating that the levels of p-p38MAPK were related to apoptosis regulation in VEGF-B-mediated cardiomyocyte protection.

Notably, several studies have suggested that p38 MAPKs regulated distinct phases of autophagy. p38MAPK can elicit autophagy via Beclin1 [26]. Interestingly, Bcl-2, a downstream regulator of PI3K/Akt, can inhibit Beclin1-dependent autophagy, and Beclin1-mediated autophagy activation during reperfusion is associated with Bcl-2 down-regulation [37]. Notably, Bcl-2 protein is also involved in the regulation of apoptosis. Decreased expression of Bcl-2 may contribute to apoptotic cell death [36]. In the present study, VEGF-B increased Bcl-2 protein levels and inhibited H9c2 cell apoptosis and autophagy while decreasing the levels of p-p38 MAPKs and Beclin1. Additionally, the specific effects could be abolished by the PI3K-Akt inhibitor LY294002 and the p38 MAPK inhibitor SB203580, demonstrating that VEGF-B prevented the detrimental effects of autophagy and apoptosis by controlling the balance between PI3K-Akt and p38 MAPK signaling.

The PI3K/Akt pathway is also linked to increased cardiomyocyte proliferation [38], and the over-expression of VEGF-B could directly induce the proliferation of resident c-kit-positive CSC in an angiotensin II-induced heart failure model [5]. In our study, we found that VEGF-B increased the number of c-kit-positive CSCs, which are associated with the activation of the PI3K/Akt pathway, in the I/R heart. In addition, MSC releases paracrine factors such as VEGF, SDF1α, and HGF. As a functional consequence, a conditioned medium of MSCs has a migratory effect on cardiac resident c-kit-positive CSCs [3]. CSCs, which express c-Kit, MDR1, and/or Sca-1 markers, are self-renewing, clonogenic and multipotent in vitro and differentiate into cardiomyocytes, smooth muscle cells, and endothelial cells in vivo. A recent study showed that endogenous c-kit-positive cells produce few new cardiomyocytes and mainly generate cardiac endothelial cells at functionally significant levels within the heart during heart development, ischemia and infarction states, suggesting that c-kit-positive cells contribute to heart repair through enhanced cardiac angiogenesis [39]. Our previous study showed that VEGF-A directly and indirectly promoted CSC mobilization in the infarcted heart via the SDF-1α/CXCR4 axis [3, 20]. In the present study, VEGF-B protein injection clearly increased the number of resident c-kit-positive CSCs in the I/R heart while inducing the expression of SDF-1α and HGF, triggering CSC mobilization, and inducing angiogenesis and vasculogenesis, leading to improved cardiac function in the I/R heart. Therefore, VEGF-B could repair the injured heart by activating the angiogenesis mediated by CSCs after long-term I/R.

## Conclusion

Taken together, the results of the present study suggest that VEGF-B can improve the short- and long-term prognosis of I/R via the dual manipulation of cardiomyocyte protection and CSCs.

## Additional files

10.1186/s12967-016-0847-3 VEGF-B suppressed CK, CK-MB and cTnT in I/R model of heart in rat in vivo. The levels of total Akt, ERK1 and p38MAPK were not obviously altered in I/R model of heart in rat in vivo and in H/R model of H9c2 cells in vitro after VEGF-B treatment. However, VEGF-B inhibited H/R-induced H9c2 cell apoptosis through up-regulating Bcl2 and down-regulating Bax expression, these specific effects could be abolished by PI3K/Akt blocker. And the representative images results showing LC3 changes were shown after SB203580 or PD98059 treatment prior to VEGF-B in different groups of H9c2 cells infected with Ad-GFP-RFP-LC3. More importantly, the long-term effects of VEGF-B on tube formation of c-kit positive CSC in vitro and heart function in vivo were shown.
10.1186/s12967-016-0847-3 Isolated c-kit+ cells from rat hearts were cultured and used to evaluate the ability of tube formation of c-Kit cells following stimulation of conditioned medium from H9c2 cells treated with VEGF-B.
