# Supplementary material for: Dual effects of VEGF-B on activating cardiomyocytes and cardiac stem cells to protect the heart against short- and long-term ischemia–reperfusion injury
Source: J Transl Med. 2016 May 4;14:116. doi: 10.1186/s12967-016-0847-3 (PMC4855341; doi:10.1186/s12967-016-0847-3)
Supplement: Supplementary file 1 — 10.1186/s12967-016-0847-3 VEGF-B suppressed CK, CK-MB and cTnT in I/R model of heart in rat in vivo. The levels of total Akt, ERK1 and p38MAPK were not obviously altered in I/R model of heart in rat in vivo and in H/R model of H9c2 cells in vitro after VEGF-B treatment. However, VEGF-B inhibited H/R-induced H9c2 cell apoptosis through up-regulating Bcl2 and down-regulating Bax expression, these specific effects could be abolished by PI3K/Akt blocker. And the representative images results showing LC3 changes were shown after SB203580 or PD98059 treatment prior to VEGF-B in different groups of H9c2 cells infected with Ad-GFP-RFP-LC3. More importantly, the long-term effects of VEGF-B on tube formation of c-kit positive CSC in vitro and heart function in vivo were shown. [file 12967_2016_847_MOESM1_ESM.docx]

**Additional file1:** VEGF-B suppressed CK, CK-MB and cTnT in I/R model of heart in rat in vivo.

The total levels of Akt, ERK1 and p38MAPK were not obviously altered in I/R model of heart in rat in vivo and in H/R model of H9c2 cells in vitro after VEGF-B treatment. However, VEGF-B inhibited H/R-induced H9c2 cell apoptosis through up-regulating Bcl2 and down-regulating Bax expression, these specific effects could be abolished by PI3K/Akt blocker. And the representative images results showing LC3 changes were shown after SB203580 or PD98059 treatment prior to VEGF-B in different groups of H9c2 cells infected with Ad-GFP-RFP-LC3. More importantly, the long-term effects of VEGF-B on tube formation of c-kit positive CSC *in vitro* and heart function *in vivo* were shown.

**Additional file 1: Figure S1.** VEGF-B suppressed CK, CK-MB and cTnT in I/R model of heart in rat *in vivo*.CK activity (A) was measured as described in Materials and Methods. CK-MB activity (B) and cTnT (C) levels were quantified by ELISA. **P* < 0.01 < 0.05 vs. sham group; ^#^*P* <0.05 vs I/R group. n = 6.**Figure S2.** VEGF-B did not change the expressions of Akt, ERK1 and p38MAPK in rat hearts following I/R. (A-D).Akt, ERK1 and p38MAPK expressions were detected by Western blot as described in Materials and Methods, and quantified by normalization to a-tubulin. **P*＞0.05 vs. Sham group; ^$^*P*＜0.05 vs. I/R-treated heart. n= 3. **Figure S3.** VEGF-B did not change the expressions Akt, ERK1 and p38MAPK in H9c2 cells following H/R. (A).Akt, ERK1 and p38MAPK expressions were detected by Western blot as described in Materials and Methods, and quantified by normalization to a-tubulin (B). **P*＞0.05 vs. 15% FBS-treated cells. n= 3.**Figure S4.**VEGF-B inhibited H/R-induced H9c2 cell apoptosis through regulating Bcl2/Bax expression mediated by PI3K/Akt signaling.(A)Akt、pAkt、Bcl-2 and Bax expressions were detected by Western blot as described in Materials and Methods, and quantified by normalization to a-tubulin(B-F).**P*< 0.05 vs. 15% FBS-treated cells; ^#^*P*< 0.05 vs. H/R-treated cells; ^&^*P*< 0.05 vs. 20 ng/ml VEGF-B-treated cells;^$^*P*＞0.05 vs. H/R-treated cells. n= 3.**Figure S5.** The effect of ERK1/2 and p38MPAK on H9c2 cells autophagy. Representative images showing LC3 staining in different groups of H9c2 cells infected with Ad-GFP-RFP-LC3 for 48 h. VEGF-B: 20ng/ml, SB was indicated as SB203580; PD was indicated as PD98059.**Figure S6.** Conditioned medium (CM) from rat cardiomyocyte line H9c2 following VEGF-B stimulation induced tube formation of c-kit positive CSC. Tube formation assay of c-kit positive CSC were performed under CM-VEGF-B or human SDF-1a (50ng/ml) with or without AMD3100 (10ug/ml) or SU11274 (10 uM) for 16 h as described in Additional file 2: Method S1.(A-F).Representative image of tube formation. (G) Quantitative analysis of tube formation assay of c-kit positive CSC. **P*< 0.05 vs. CM-CTRL;^#,$^*P*< 0.05 vs. CM-VEGF-B; ^&^*P*< 0.05 vs.CM-CTRL;^$^*P*＞0.05 vs.CM-CTRL+hSDF-1. n= 3**.Figure S7.** VEGF-B improved heart function.LV function was measured as described in Materials and Methods. LVSP-left ventricle systolic pressure (A); LVEDP-left ventricle end-diastolic pressure (B); ± d*p*/d*t*_max_-rate of the rise or fall of left ventricular pressure (C and D). ^&^*P* < 0.05 vs. sham group; ^#,&^*P* < 0.05 vs. I/R group; ^$^*P* < 0.01 vs. VEGF-B 1.0 injection group. n = 6.

**Figure S1. VEGF-B suppressed CK, CK-MB and cTnT in I/R model of heart in rat *in vivo***

**
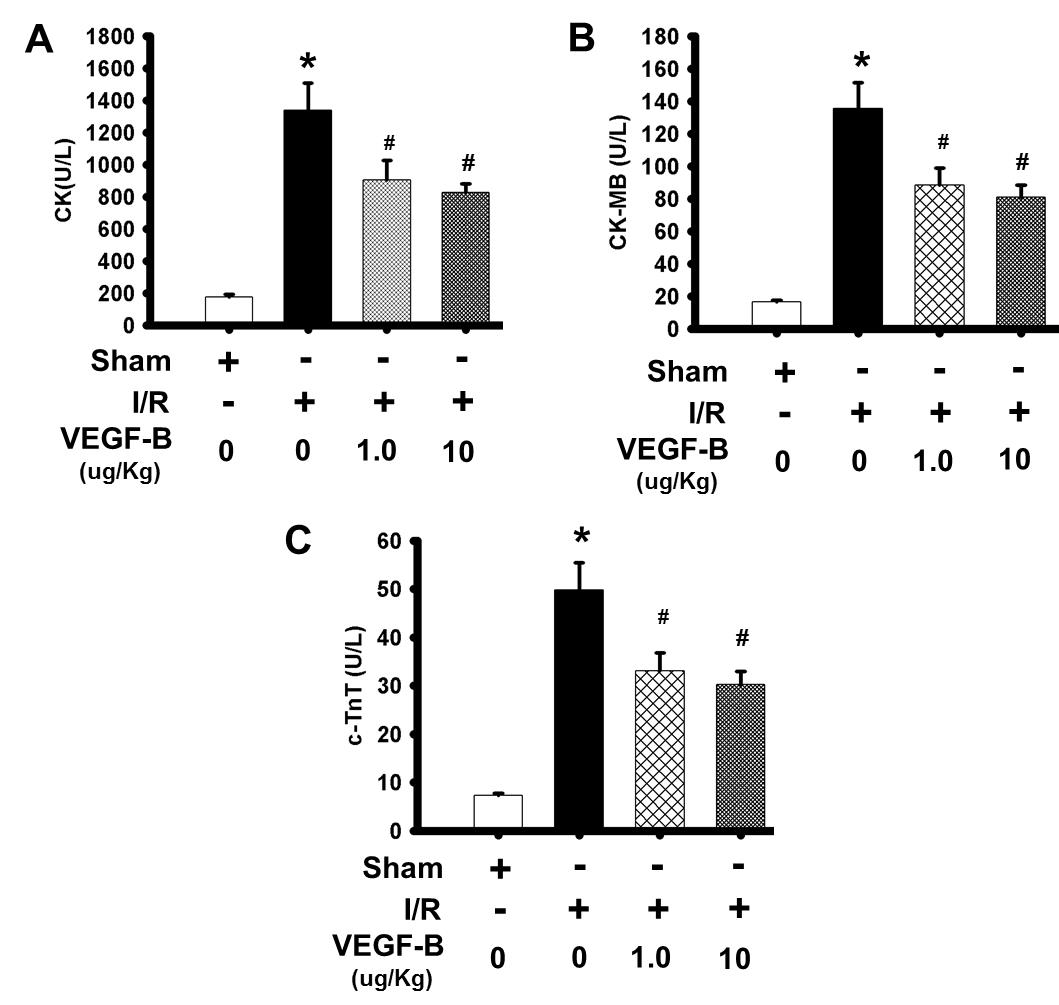
**

**Figure S2. VEGF-B did not change the expressions Akt, ERK1 and p38MAPK in rat hearts following I/R**


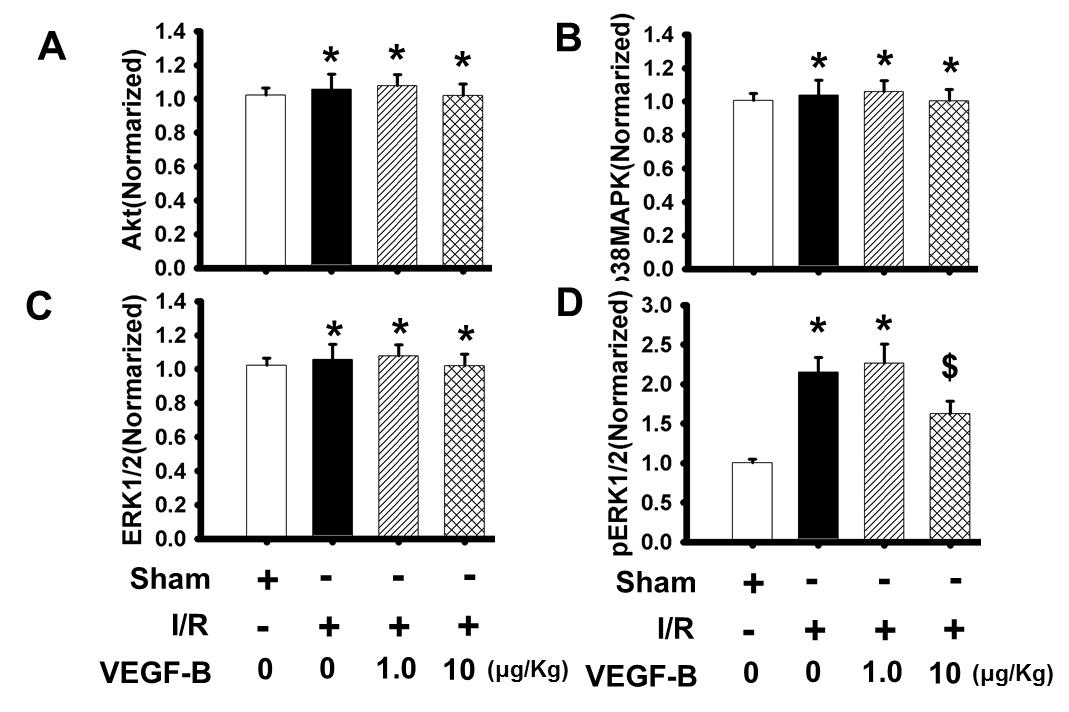


**Figure S3.VEGF-B did not change the expressions Akt, ERK1 and p38MAPK in H9c2 cells following H/R**


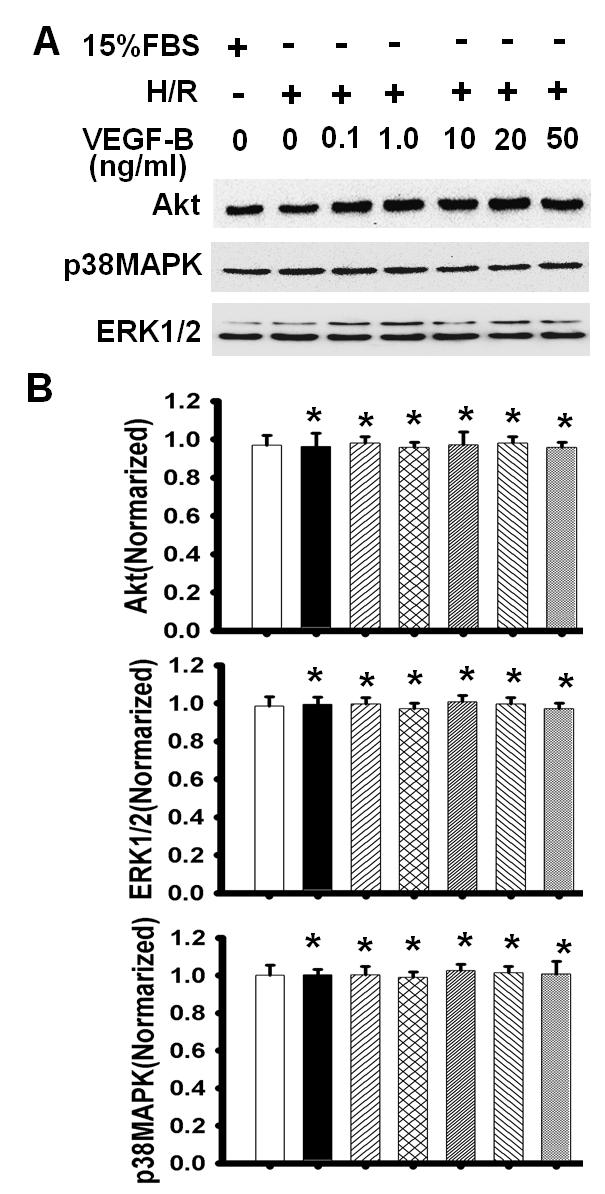


**Figure S4.VEGF-B inhibited H/R-induced H9c2 cell apoptosis through regulating Bcl2/Bax expression mediated by PI3K/Akt signaling**


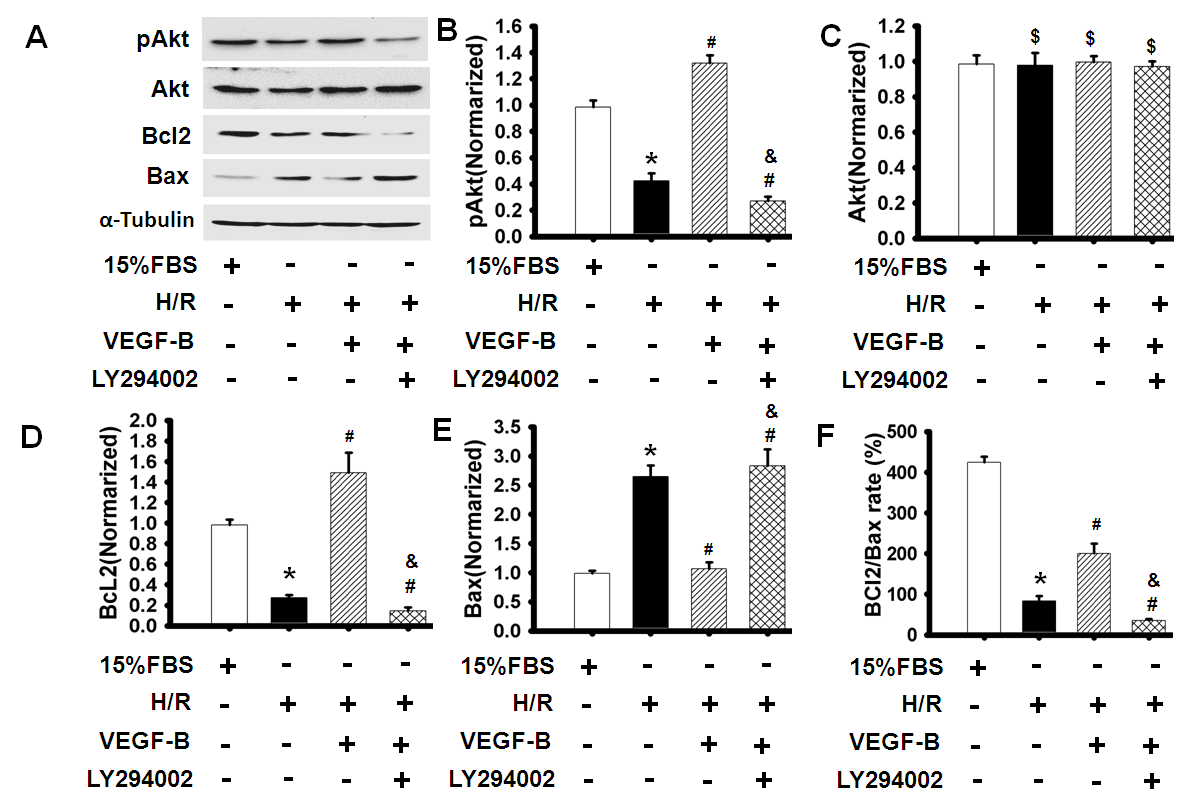


**Figure S5. The effect of ERK1/2 and p38MPAK on H9c2 cells autophagy**


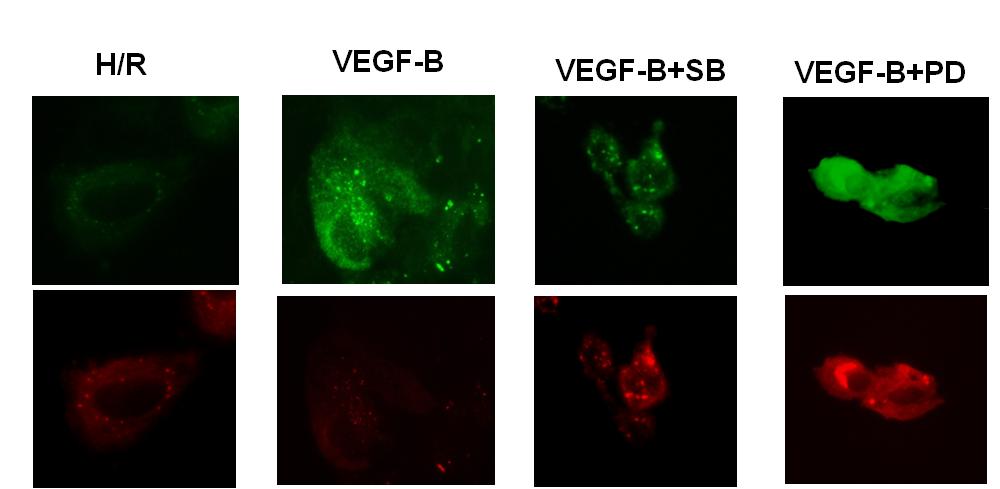


**Figure S6. Conditioned medium (CM) from rat cardiomyocyte line H9c2 following VEGF-B stimulation induced tube formation of c-kit positive CSC**

**
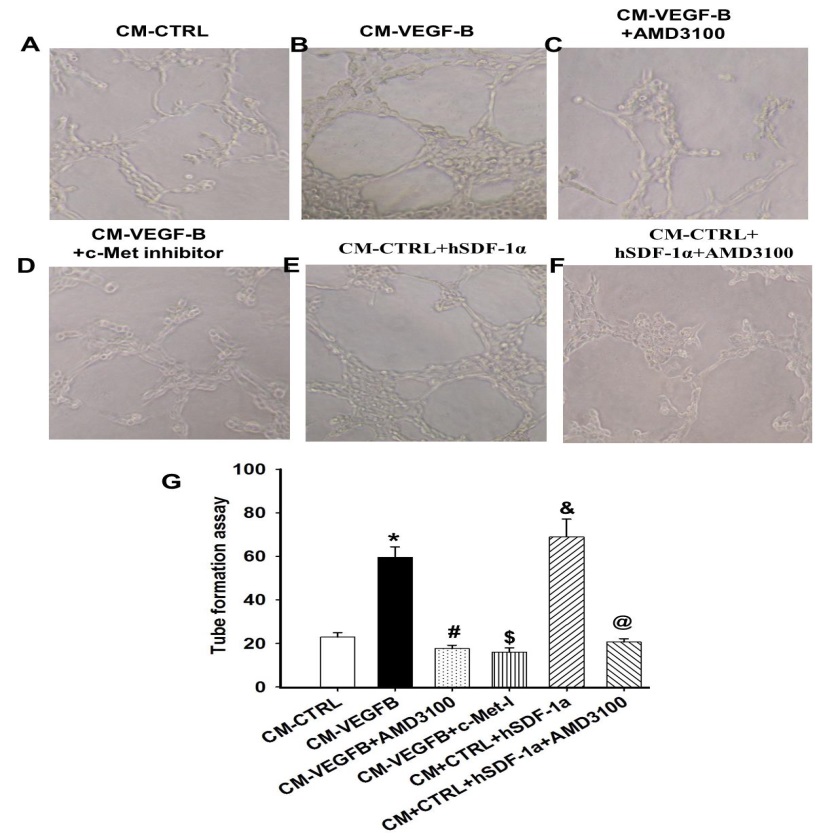
**

**Figure S7. VEGF-B improved heart function**


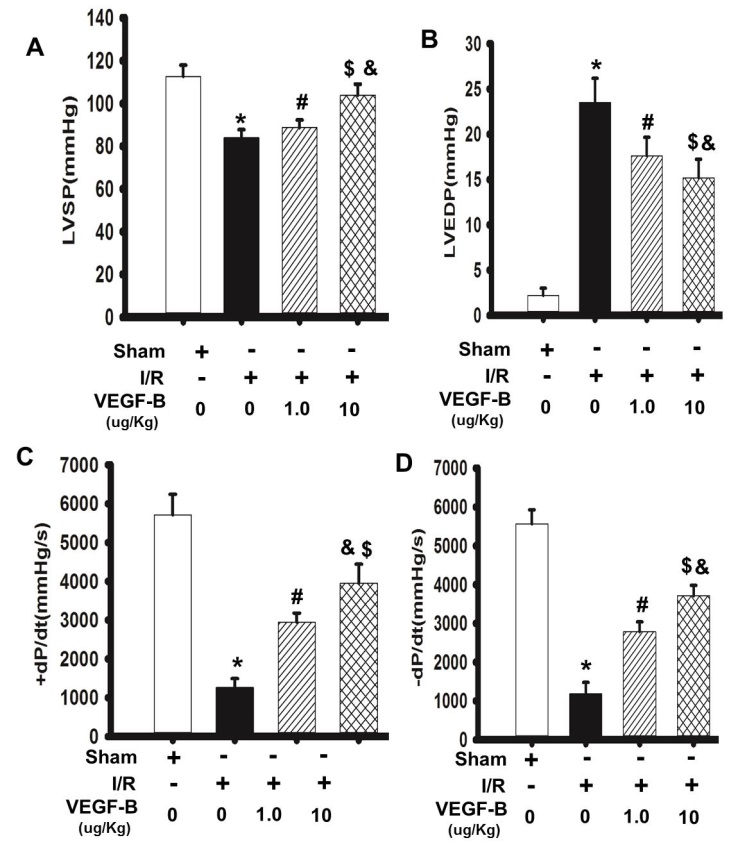


**Abbreviation**

VEGF: vascular endothelial growth factor. CSC: cardiac stem cell. H/R: Hypoxia-reoxygenation. I/R: ischemia-reperfusion injury. MI: Myocardial infarction. IHD: ischemic heart disease. PCI: percutaneous coronary intervention. LAD: left anterior descending coronary artery. CK: creatine kinase. CTnT: cardiac troponin T. PBS: phosphate-buffered saline. LDH: Lactate dehydrogenase.CXCR4: C-X-C chemokine receptor type4.SDF-1α: stromal cell-derived factor-α. HGF: hepatocyte growth factor. MAPK: mitogen-activated protein kinases. ERK: extracellular-signal related kinase. PI3K: phosphatidylinositol 3-kinase. Akt: RAC-alpha serine/threonine-protein kinase. Bcl2: B-cell lymphoma-2. LVSP: left ventricle systolic pressure. LVEDP: left ventricle end-diastolic pressure.± d*p*/d*t*_max_: rate of the rise or fall of left ventricular pressure.
