# Supplementary material for: Dual effects of VEGF-B on activating cardiomyocytes and cardiac stem cells to protect the heart against short- and long-term ischemia–reperfusion injury
Source: J Transl Med. 2016 May 4;14:116. doi: 10.1186/s12967-016-0847-3 (PMC4855341; doi:10.1186/s12967-016-0847-3)
Supplement: Supplementary file 2 — 10.1186/s12967-016-0847-3 Isolated c-kit+ cells from rat hearts were cultured and used to evaluate the ability of tube formation of c-Kit cells following stimulation of conditioned medium from H9c2 cells treated with VEGF-B. [file 12967_2016_847_MOESM2_ESM.doc]

**Additional file2:** Isolated c-kit+ cells from rat hearts were cultured and used to evaluate the ability of tube formation of c-Kit cells following stimulation of conditioned medium from H9c2 cells treated with VEGF-B.

**Additional file2: Method S1.** Isolation and culture of c-kit+ cells from rat hearts.

CSCs were isolated from the hearts of 3-day-old Sprague-Dawley rats with a method described by Beltrami *et al* [1, 2] with a minor modification. Briefly, the hearts were removed under aseptic conditions from rats that were overdosed with sodium pentobarbital. The myocardial tissue was cut into 1-2 mm^3^pieces,washed with Ca^2+^-Mg^2+^-free phosphate-buffered solution(PBS) to remove the blood, and then digested with 0.2% trypsin (Invitrogen) and 0.1% collagenase IV (Sigma, Milan, Italy) three times for 5 minutes each at37°C. After the enzymatic digestion, cell suspension was collected and filtered with a strainer (Becton Dickson). The cells were then incubated with rabbit anti-c-kit antibody (Santa Cruz) and separated using sheep anti-rabbit immunomagneticmicrobeads (MiltenyiBiotec, Auburn, CA). Small round cells positive for c-kit antibody were collected. Newly isolated cardiac c-kit^+^ cells were grown in fibronectin-coated 25 cm^2^ culture flasks in DMEM containing 15% fetal calf serum (FCS, Hyclone, USA), 10 ng/mL bFGF and 10 ng/mL LIF for 3 days (5% CO_2_, 37°C). After recovery, the cells were used for the experiments. Toinduce differentiation, cells were cultured in medium containing15% FCS and 50ng/mL VEGF (Upstate) in 24-well plates for 7 days.

**Method S2.** Conditioned medium (CM) preparation.

Rat cardiomyocyte line H9c2 was obtained from the Cell Bank of the Shanghai Institutes for Biological Sciences, Chinese Academy of Sciences (SIBS CAS, China).Conditioned medium (CM) from H9c2 was generated as follows[3]: for purpose of normalization, 3×10^6^ rat cardiomyocyte line H9c2 were cultured in 75 cm^2^ Flask for 24h in a complete medium. And then the cells were cultured for 24h in new complete medium with (CM-VB) or without (CM-Ctrl) 20ng/ml VEGF-B at 37 ℃ with 5% of CO_2_. The culture medium was then collected, centrifuged and filtrated for in vitro experiments.

**Method S3.** c-Kit cells-derived tube formation assay.

Tube formation assays were performed as described [4]. Briefly, c-Kit cells (4×10^5^/ml) were seeded in 96-well plates coated with MatrigelTM and incubated in conditioned medium indicated in Method S1. Where indicated, CXCR4 inhibitor AMD3100 (10ug/ml, Sigma, USA) or c-Met inhibitor SU11274(10Um,Calbiochem，USA) treatments was done 30 minutes prior to changing to CM-VB, AMD3100-supplemented CM-Ctrl with 50 ng/ml recombinant human SDF-1α. For this assay, 3–4 wells/ group were used and the total number of tubes per well was quantified and averaged. Cells were incubated at 37°C for 16 h, fixed in formalin, washed with PBS, and imaged using microscopy. Number of tube branches (in pixels) was quantified using ImageJ software (NIH).

**Abbreviation**

VEGF-B: vascular endothelial growth factor B. CSC: cardiac stem cell. CM: Conditioned medium. CM-VB: Conditioned medium after treatment with VEGF-B. CM-Ctrl: Conditioned medium after treatment without 20ng/ml VEGF-B. PBS: phosphate-buffered solution. FCS: fetal bFGF: basic fibroblast growth factor. LIF: leukocyte inhibitory factor. c-Met: hepatocyte growth factor receptor.CXCR4: C-X-C chemokine receptor type4. SDF-1α: stromal cell-derived factor-α.HGF: hepatocyte growth factor. DMEM: Dulbecco minimum essential medium.

**References:**

[1]. A.P. Beltrami, L. Barlucchi, D. Torella, M. Baker, F. Limana, S. Chimenti, H. Kasahara, M .Rota, E. Musso, K. Urbanek, A. Leri, J. Kajstura, B. Nadal-Ginard, P. Anversa, Adult cardiac stem cells are multipotent and support myocardial regeneration, Cell. 114 (2003) 763-776.

[2]. Tang J, Wang J, Kong X, Yang J, Guo L, Zheng F, Zhang L, Huang Y, Wan Y.Vascular endothelial growth factor promotes cardiac stem cell migration via the PI3K/Aktpathway.Exp Cell Res. 2009 ;315(20):3521-31.

[3]Tang JM, Wang JN, Zhang L, Zheng F, Yang JY, Kong X, Guo LY, Chen L, Huang YZ, Wan Y, Chen SY. VEGF/SDF-1 promotes cardiac stem cell mobilization and myocardial repair in the infarcted heart. *Cardiovasc Res*. 2011; 91:402-11.

[4]Chen JK, Deng YP, Jiang GJ, Liu YZ, Zhao T, ShenFM.Establishment of tube formation assay of bone marrow-derived endothelial progenitor cells.CNS Neurosci Ther. 2013;19(7):533-5.
